# Perinatal exposure to the autism-linked metabolite *p*-Cresol has limited impact on early development in mice but lasting effects on adult social behavior

**DOI:** 10.1038/s41598-025-96840-8

**Published:** 2025-04-15

**Authors:** Juliette Canaguier, Geoffroy Mallaret, Cristina Paraschivescu, Susana Barbosa, Julie Le Merrer, Jérôme Becker, Nicolas Glaichenhaus, Laetitia Davidovic

**Affiliations:** 1 Université Côte d’Azur, CNRS UMR7275, INSERM U1318, Institut de Pharmacologie Moléculaire et Cellulaire, Valbonne, France; 2 iBrain, UMR 1253, Université de Tours, Inserm, CNRS, Tours, France; 3 Alliance FondaMental, Créteil, France; 4 Present Address: Center for Immunology and Inflammation, The Feinstein Institute for Medical Research, Manhasset, NY USA; 5 Present Address: Institut Pierre Louis d‘Épidémiologie et de Santé Publique, Inserm, Paris, France

**Keywords:** Neuroscience, Autism spectrum disorders

## Abstract

Environmental exposures during pregnancy are increasingly recognized as significant risk factors for the offspring health. Perinatal exposure to environmental toxins, including *p-*Cresol, may disrupt essential physiological processes during critical developmental windows. The perinatal period is particularly critical for neurodevelopment, making the brain particularly vulnerable to external toxins that can impair neural circuitry and increase the risk of behavioral and neuropsychiatric disorders later in life. *p*-Cresol, a microbial metabolite produced by the human microbiota and through the bacterial decomposition of organic matter, is also released in exhaust fumes and widely used in chemical manufacturing. Elevated levels of *p*-Cresol have been consistently observed in patients with autism spectrum disorders (ASD), yet, its developmental toxicity and its impact on ASD-related behaviors remain to be determined. In this study, we exposed pregnant mice (C57BL/6J) to *p-*Cresol via drinking water from mid-gestation, through lactation, and until weaning, mimicking potential patterns of human exposure during pregnancy and early infancy. Our findings indicated that while perinatal *p*-Cresol exposure did not affect gestational outcomes, postnatal growth, developmental milestones, or sensorimotor reflexes, it resulted in marked social deficits and stereotyped behaviors in adult males and females offspring—which are core behavioral phenotypes associated with ASD. These results suggest that *p*-Cresol exposure selectively induces ASD-like behaviors, without broadly impairing early development. Our findings emphasize the need for future epidemiological studies to quantify *p*-Cresol exposure during pregnancy and early childhood, and to evaluate its potential association with social behavior impairments in children. This research provides a foundation for understanding how environmental factors, such as *p*-Cresol, may contribute to social impairments and neurodevelopmental disorders in the broader population.

**Supplementary Information:**

The online version contains supplementary material available at 10.1038/s41598-025-96840-8.

## Introduction

The metabolite *p-*Cresol (also known as 4-cresol, 4-methylphenol) is an alkylphenolic compound commonly found in the environment, due to both natural processes and industrial activities. *p*-Cresol is naturally produced by the microbial decomposition of organic matter and notably by the human gut microbiota. Certain gut bacteria, such as *Clostridioides difficile* or *Escherichia coli* metabolize the dietary aromatic amino acid tyrosine into *p*-Cresol^1,2^. Foods rich in phenolic compounds, particularly those containing high levels of tyrosine, can serve as precursors for *p*-Cresol formation. Protein-rich diets, including the consumption of meat and dairy products, have been linked to higher levels of *p*-Cresol production due to the increased availability of tyrosine for microbial fermentation^3^. Additionally, certain processed foods, such as cured meats and smoked fish, contain phenolic compounds, including *p*-Cresol, due to the use of preservatives or smoking methods^4^. Diet therefore contributes to overall *p*-Cresol exposure both via internal microbiota production and direct intake of *p*-Cresol. *p-*Cresol is also widely used in the production of industrial chemicals, including antioxidants, disinfectants, resins, paints, and pharmaceuticals^5,6^. *p-*Cresol was originally extracted from coal and beech tar but can now be produced synthetically through the methylation of phenol. Industrial and environmental contamination with *p*-Cresol occurs through pathways such as agricultural runoff from herbicides and pesticides, emissions from fossil fuel combustion, and effluent discharge from wastewater treatment plants^7^. Finally, *p-*Cresol is one of the main photooxidation products of toluene, the most abundant aromatic hydrocarbon in the atmosphere emitted primarily from automobiles and industrial activities^8^. These multiple natural and environmental sources contribute to its presence in air, water, and soil, raising concerns about the extent of human environmental exposure.

The health risks associated with *p*-Cresol have been documented mostly in rodents^9^. No significant adverse health effects were reported in mice following chronic low dose exposure to *p-*Cresol when dispensed at 50 mg/kg/day by daily oral gavage^10^ or through drinking water to mimic chronic gastro-Intestinal (GI) exposure^11^. Toxicological studies report that mice chronically exposed to high doses of *p-*Cresol (> 500 mg/kg/day orally gavaged once a day) exhibited liver and kidney damage, as well as neurological issues (hypoactivity, tremors), with no noticeable effect on the gastro-intestinal tract^9^. Regulatory agencies (e.g. European Chemicals Agency (ECHA), US Occupational Safety and Health Administration (OSHA)), have implemented guidelines for controlling *p*-Cresol exposure in occupational and environmental settings. OSHA has set a permissible occupational exposure limit for *p*-Cresol at 5 ppm (22 mg/m³) daily per 8-hour workday to minimize health risks associated with prolonged inhalation. ECHA recommends labeling for skin and eye irritation hazards and protective measures during handling. *p-*Cresol is considered toxic to aquatic life, necessitating environmental precautions to prevent industrial discharges and pollution. While *p*-Cresol enters water sources, it is not commonly detected at high levels in treated drinking water due to standard purification processes which remove organic contaminants. ​Despite these regulatory efforts, the compound’s presence in consumer products, indoor environments, drinking water, diet, as well as a microbial byproduct in the human body increases the risk of chronic low-level exposure, especially in vulnerable populations.

Recent research has highlighted a significant connection between *p*-Cresol and autism spectrum disorder (ASD), as elevated levels of *p*-Cresol have been observed in the urine and stool of ASD patients, likely due to increased abundances of bacterial *p*-Cresol producers such as *Clostridia*^12–17^. ASD core symptoms encompass social interaction and communication deficits, perseverative/stereotyped behaviors, restricted interests, and abnormal sensory processing^18^. We have previously exposed male mice for 4 weeks (from 4.5 weeks old to adulthood) to low doses of *p-C*resol dispensed in drinking water (50 mg/kg/day)^11^. While this treatment did not alter growth, drink and food consumption, we showed that it selectively induced social behavior deficits, stereotypies, and perseverative behaviors, reminiscent of ASD core symptoms, with no impact on locomotor activity or cognition^11^. Studies suggest that perinatal exposure to various environmental toxins, including phenolic compounds like *p-*Cresol, can interfere with critical developmental processes, leading to long-term cognitive and behavioral changes^19^. The perinatal period is a time of heightened sensitivity, as the developing brain is particularly vulnerable to environmental insults that can disrupt neural pathways and synaptic plasticity. However, it remains to be determined whether early perinatal *p*-Cresol exposure could disrupt early neurodevelopmental processes and impair behavior.

**Fig. 1:**
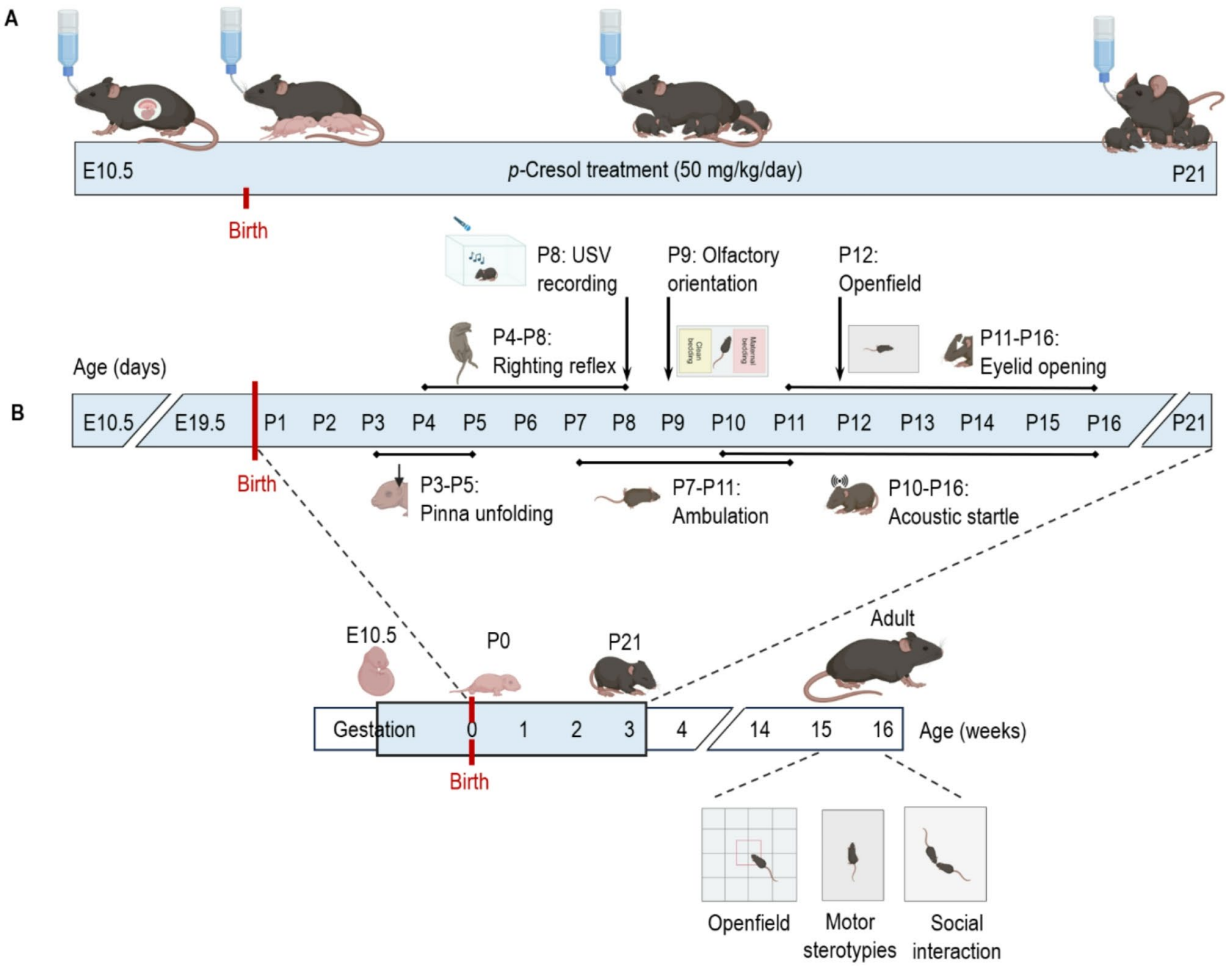
Experimental design and timeline. (**A**) Timeline of *p*-Cresol treatment in maternal drinking water from E10.5 til weaning (P21). (**B**) Offspring behavioral phenotyping: in the perinatal period (from P3 to P16) and in adulthood (15-week-old, i.e. 3.5-month-old).

In this study, pregnant female mice were exposed to *p-*Cresol via drinking water from mid-gestation through weaning, mimicking the exposure patterns that might occur in humans through environmental, microbiota, and dietary sources. Male and female offspring from *p-*Cresol exposed or unexposed mothers were longitudinally evaluated in the early postnatal period for developmental milestones acquisition, sensorimotor reflexes ontogeny, and behavioral development, as well as behavioral changes in adulthood (Fig. 1A, B).

## Results

### Perinatal exposure to *p*-Cresol does not impact the overall growth, or the acquisition of developmental milestones and sensorimotor reflexes

*p*-Cresol exposure from embryonic day 10.5 (E10.5) did not impact the body weight gain, drink or food intake of pregnant dams (Fig. S1A-D, Tab. S1). Based on the mean drink consumption and body mass at E14.5 and E17.5, we estimated daily *p-*Cresol uptake was 42-53.5 mg/kg/day (cf. Methods), in agreement with the no observed effect levels reported for *p-*Cresol^10^. All dams gave birth between E19 and E19.5 and no changes were observed in the average litter size at birth, pup survival rate at P3, litter composition, or sex ratio prior to randomized cross-fostering at P3 between *p-*Cresol-treated and control pups (Tab. S1, Fig. S1E).

**Fig. 2:**
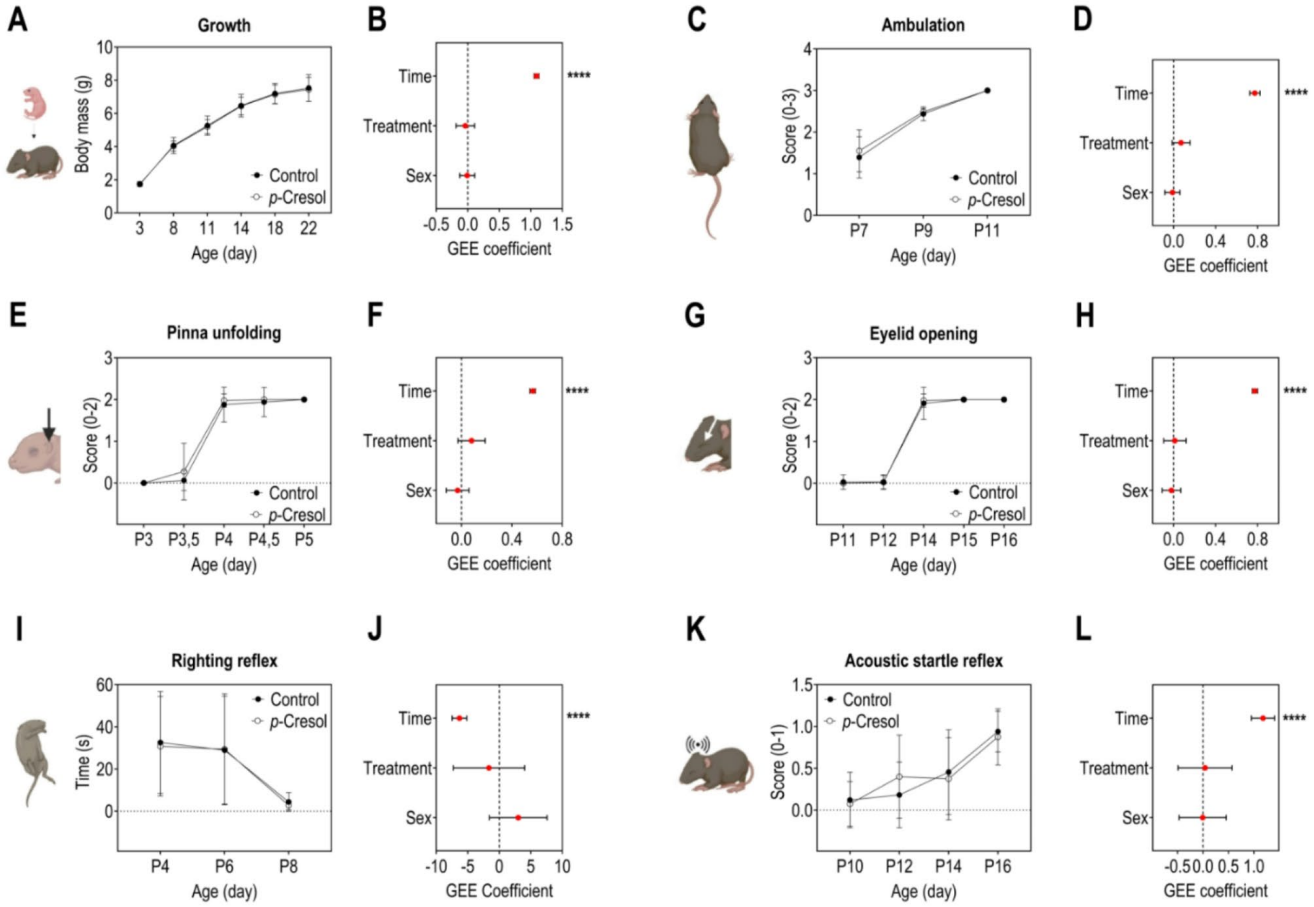
Perinatal exposure to *p*-Cresol does not impact growth, developmental milestones, and sensorimotor reflexes acquisition in mice pups. (**A**) Monitoring of body weight (P3 to P22). (**B**) GEE estimates, 95% CIs, and associated p-values for the effects of time, treatment, and sex on body weight: p(Treatment) = 0.6093, p(Time) < 0.0001, p(Sex) = 0.8885. (**C**) Ambulation rated from 0 to 3 (P7 to P11). (**D**) GEE estimates, 95% CIs, and associated p-values for the effects of time, treatment, and sex on ambulation: p(Treatment) = 0.1045, p(Time) < 0.0001, p(Sex) = 0.7380. (**E**) Pinna unfolding (P3 to P5): a score of 0, 1, or 2 was assigned based on the number of unfolded ears. (**F**) GEE estimates, 95% CIs, and associated p-values for the effects of time, treatment, and sex on ear development: p(Treatment) = 0.1405, p(Time) < 0.0001, p(Sex) = 0.5167. (**G**) Eyelid opening (P11 to P16): a score of 0, 1, or 2 was assigned based on the number of open eyelids. ( **H**) GEE estimates, 95% CIs, and associated p-values for the effects of time, treatment, and sex on eyelid opening: p(Treatment) = 0.8226, p(Time) < 0.0001, p(Sex) = 0.6393. (**I**) Righting reflex latency to turn over (P4 to P8). (**J**) GEE estimates, 95% CIs, and associated p-values for the effects of time, treatment, and sex on the righting reflex: p(Treatment) = 0.5701, p(Time) < 0.0001, p(Sex) = 0.1960. (**K**) Acoustic startle reflex (P10 to P16): score of 0 or 1, with 1 assigned to pups that startle and 0 to those that do not startle. (**L**) GEE estimates, 95% CIs, and associated p-values for the effects of time, treatment, and sex on the acoustic startle reflex: p(Treatment) = 0.8789, p(Time) < 0.0001, p(Sex) = 0.9805. (**A**, **C**, **E**, **G**, **I**, **K**) Data are presented as means ± standard deviation. (**B**, **D**, **F**, **H**, **J**, **L**) Data are presented as dot plots with means ± standard deviation. ****p < 0.0001. Only statistically significant differences are presented (*p* < 0.05). (**A–L**) *n* = 33 control (22 males/11 females), *n* = 40 *p*-Cresol (18 males/22 females).

During the first three weeks of postnatal life, we monitored longitudinally developmental milestones, sensorimotor reflexes, and behavioral development of both male and female offspring. To simultaneously assess the effects of time, treatment, and sex on each outcome, we used generalized estimating equations (GEE), a multivariable statistical approach (Fig. 2). While GEE revealed a significant effect of time on pups’ growth (Fig. 2A, B) and developmental milestones acquisition: ambulation (Fig. 2C, D), pinna unfolding (Fig. 2E, F), and eyelid opening (Fig. 2G, H), there was no significant effect of *p*-Cresol treatment or sex. Similarly, a significant effect of time was noted on sensorimotor reflexes acquisition: righting reflex (Fig. 2I, J) and acoustic startle reflex (Fig. 2K, L), while no significant effect of *p*-Cresol treatment or sex were observed. These results suggest that *p*-Cresol perinatal exposure *via* the mother does not interfere with offspring’s growth, nor does it induce developmental delays, or impact sensorimotor reflex acquisition during early life.

### Perinatal exposure to *p*-Cresol impairs communication and reduces locomotor activity

**Fig. 3:**
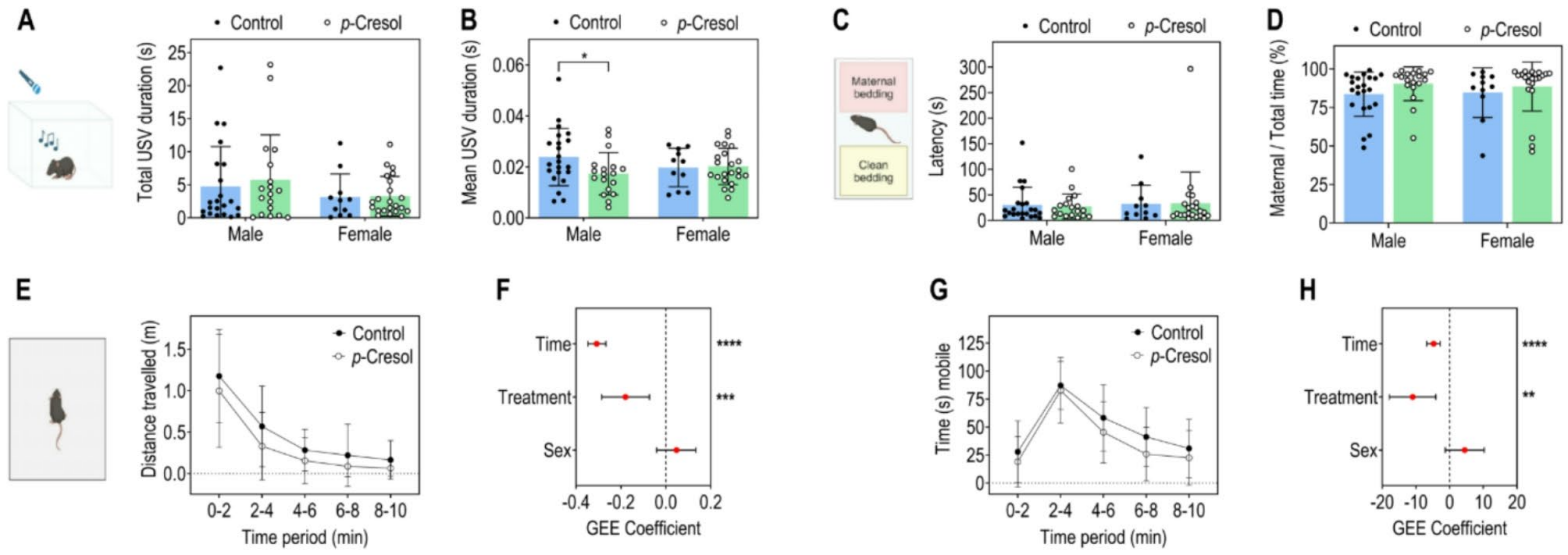
Perinatal exposure to *p*-Cresol impairs communication in male mice pups and induces hypoactivity in mice pups, with no changes in olfactory orientation. (**A**) Total duration of USV emitted in male and female pups at P8; two-way ANOVA: p(Treatment) = 0.6643, p(Sex) = 0.1106, p(Treatment x Sex) = 0.7332; Šidák post hoc tests for treatment effect by sex: *p* > 0.05. (**B**) Mean USV duration; two-way ANOVA: p(Treatment) = 0.1627, p(Sex) = 0.7696, p(Treatment x Sex) = 0.1125; Šidák post hoc tests for treatment effect by sex: male *p < 0.05. (**C**) Latency to the first entry into the maternal bedding zone in the olfactory orientation test at P9; two-way ANOVA: p(Treatment) = 0.9385, p(Sex) = 0.6904, p(Treatment x Sex) = 0.8331; Šidák post hoc tests for treatment effect by sex: *p* > 0.05. (**D**) Ratio between time spent in maternal zone vs. total time; two-way ANOVA: p(Treatment) = 0.1312, p(Sexe) = 0.9103, p(Treatment x Sexe) = 0.6962; Šidák post hoc tests for treatment effect by sex: *p* > 0.05. (**E**) Distance traveled over time (2-minute bins) at P12. **F.** GEE estimates, 95% CIs, and associated p-values for the effects of time, treatment, and sex on distance traveled: p(Treatment) = 0.0009, p(Time) < 0.0001, p(Sex) = 0.3004. (**G**) Time spent in motion (2-minute bins). (**H**) GEE estimates, 95% CIs, and associated p-values for the effects of time, treatment, and sex on time spent in motion: p(Treatment) = 0.0019, p(Time) < 0.0001, p(Sex) = 0.1272. (**A–D**) Data are presented as dot plots indicating means ± standard deviation. (**E**, **G**) Data are presented as means ± standard deviation. (**F**, **H**) Data are presented as dot plots with means ± standard deviation. *p < 0.05, **p < 0.01, ***p < 0.001, ****p < 0.0001. Only statistically significant differences are presented (*p* < 0.05). (**A–H**) *n* = 33 control (22 males/11 females), *n* = 40 *p*-Cresol (18 males/22 females).

We then studied communication abilities in the offspring of *p*-Cresol-treated or control mice by monitoring the USV emitted by each pup at P8, after a brief separation from the mother. There was no difference in the loss of body temperature during the USV test (Fig. S2F), suggesting that *p*-Cresol perinatal exposure did not induce changes in thermoregulation, which could interfere with USV measures. The total duration and number of USV emitted were similar between the offspring of *p*-Cresol-treated and untreated mice, with no sex effect (Fig. 3A, Fig. S2E). However, a small, yet significant, decrease in USV mean duration was observed specifically in the male offspring of *p-*Cresol-treated mice (Fig. 3B). These findings suggest that *p-*Cresol perinatal exposure has subtle effects on early communication, only observable in male pups.

In the olfactory orientation test at P9, mouse pups exposed to *p*-Cresol and those in the control group showed similar behavior in terms of latency to reach the maternal bedding (Fig. 3C), ratio of time spent in maternal bedding vs. total time (Fig. 3D), and first choice to reach maternal bedding (Fig. S2A, B). Consistently, *p*-Cresol treatment had no effect on the number of entries in each zone (Fig. S2C, D). This indicates that, at this stage, pups adequately detect maternal olfactory cues and exhibit maternal attachment.

When monitoring locomotor activity at P12, compared to control pups, *p*-Cresol-exposed pups covered shorter distances (Fig. 3E, F), indicating hypoactivity. Furthermore, they spent less time in motion (Fig. 3G, H) than control pups, especially in the latest phase of the test (period 4–10 min, Fig. 3G). There was a strong effect of time but no effect of sex on either of these parameters.

### Perinatal exposure to *p-*Cresol selectively induces motor stereotypies and social behavior deficits in adult mice

**Fig. 4:**
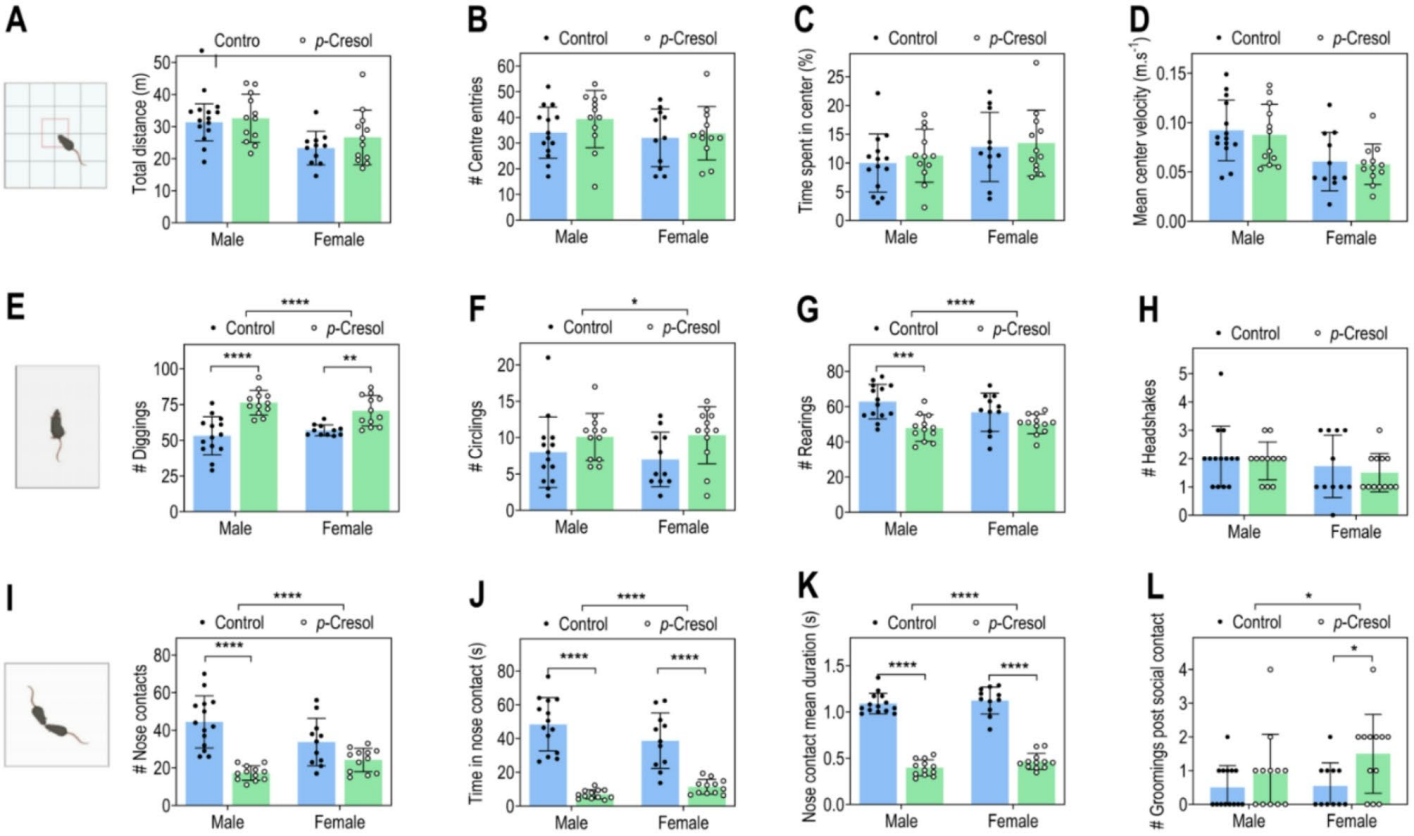
Perinatal exposure to *p*-Cresol does not induce anxiety or impair locomotor activity, but partially induces motor stereotypies and impairs social behavior in adult mice. (**A**) Total distance traveled in the open-field test; two-way ANOVA: p(Treatment) = 0.2504, p(Sex) = 0.0009, p(Treatment x Sex) = 0.6020; Šidák post hoc tests for treatment effect by sex: *p* > 0.05. (**B**) Number of entries into the center zone; two-way ANOVA: p(Treatment) = 0.2517, p(Sex) = 0.2218, p(Treatment x Sex) = 0.5775; Šidák post hoc tests for treatment effect by sex: *p* > 0.05. (**C**) Percentage of time spent in the center; two-way ANOVA: p(Treatment) = 0.5304, p(Sex) = 0.1114, p(Treatment x Sex) = 0.8465; Šidák post hoc tests for treatment effect by sex: *p* > 0.05. (**D**) Average speed in the center zone; two-way ANOVA: p(Treatment) = 0.6616, p(Sex) = 0.0005, p(Treatment x Sex) = 0.9054; Šidák post hoc tests for treatment effect by sex: *p* > 0.05. (**E**) Number of diggings; two-way ANOVA: p(Treatment) < 0.0001, p(Sex) = 0.7660, p(Treatment x Sex) = 0.1084; Šidák post hoc tests for treatment effect by sex: Male *p* < 0.0001 ; female *p* = 0.0043. (**F**) Number of circlings; two-way ANOVA: p(Treatment) = 0.0235, p(Sex) = 0.7469, p(Treatment x Sex) = 0.5910 ; Šidák post hoc tests for treatment effect by sex: *p* > 0.05. (**G**) Number of rearings; two-way ANOVA: p(Treatment) < 0.0001, p(Sex) = 0.4589, p(Treatment x Sex) = 0.0993; Šidák post hoc tests for treatment effect by sex: Male *p* < 0.001 ; female *p* > 0.05. (**H**) Number of headshakes; two-way ANOVA: p(Treatment) = 0.4666, p(Sex) = 0.1506, p(Treatment x Sex) = 0.8898; Šidák post hoc tests for treatment effect by sex: *p* > 0.05. (**I**) Number of nose contacts; two-way ANOVA: p(Treatment) < 0.0001, p(Sex) = 0.5212, p(Treatment x Sex) = 0.0043; Šidák post hoc tests for treatment effect by sex: Male *p* < 0.0001 ; female *p* > 0.05. **J.** Total time spent in nose contact; two-way ANOVA: p(Treatment) < 0.0001, p(Sex) = 0.4433, p(Treatment x Sex) = 0.0411; Šidák post hoc tests for treatment effect by sex: *p* < 0.0001. (**K**) Mean duration of one nose contact; two-way ANOVA: p(Treatment) < 0.0001, p(Sex) = 0.1085, p(Treatment x Sex) = 0.5635; Šidák post hoc tests for treatment effect by sex: *p* < 0.0001. (**L**) Number of grooming post-social interaction; two-way ANOVA: p(Treatment) = 0.0149, p(Sex) = 0.2519, p(Treatment x Sex) = 0.3261; Šidák post hoc tests for treatment effect by sex: male *p* > 0.05 ; female *p* < 0.05. (**A–L**) Data are presented as dot plots with means ± standard deviation. *p < 0.05, **p < 0.01, ***p < 0.001, ****p < 0.0001. Only statistically significant differences are presented (*p* < 0.05). (**A–L**) *n* = 25 control (14 males/11 females), *n* = 24 *p-*Cresol (12 males/12 females).

After weaning at P21, male and female pups were separated from *p*-Cresol-treated or control mothers, grouped by sex in cages with *ad libitum* access to regular drinking water and chow, and left undisturbed until adulthood (3.5-month-old). Perinatal *p-*Cresol exposure did not impact the bodyweight of adult mice, regardless of sex (Fig. S3A). In the open-field test, we observed that perinatal *p-*Cresol exposure had no discernible impact on the total distance travelled, number of entries, time spent, and average velocity in the center (Fig. 4A-D). These data show that perinatal *p-*Cresol exposure does not alter locomotor activity, nor does it induce anxiety in adult mice.

Direct monitoring of motor stereotypies revealed an overall effect of perinatal *p*-Cresol exposure on the number of diggings, circlings, and rearings, with significantly more diggings in both males and females and significantly less rearings in males, possibly indicative of diminished vertical activity (Fig. 4E-G). Conversely, there was no impact on the number of headshakes or groomings and their mean duration (Fig. 4H, Fig. S3B, C).

When monitoring social abilities in the dyadic social interaction test, there was a general effect of perinatal *p-*Cresol exposure on the number, time spent and mean duration of nose contacts, and on the number of groomings post social interaction, a sign of discomfort in mice (Fig. 4I-L). More specifically, male mice perinatally exposed to *p*-Cresol made significantly less nose contacts (Fig. 4I). Also, regardless of sex, they spent less time engaged in nose contacts compared to the control group (Fig. 4J). The average duration of these contacts was also reduced (Fig. 4K) and there was a significant increase in the number of groomings post-social interaction in females perinatally exposed to *p-*Cresol (Fig. 4L). These findings indicate that perinatal *p-*Cresol exposure via the mother has lasting impact on offspring social abilities in adulthood, with marked social interaction deficits, both in male and female offspring.

## Discussion

In this study, mice were exposed to *p*-Cresol via drinking water from mid-gestation until weaning, mimicking the exposure patterns that might occur in humans during pregnancy and infancy. The exposure route used corresponds to relevant human exposure scenarios since it ensures continuous exposure from mid-gestation through lactation and possible transmission to the offspring via the placenta during pregnancy and maternal milk after birth. Dispensing *p*-Cresol in drinking water at the dose used (50 mg/kg/day) did not induce overt toxicity in dams and appeared safe with respect to reproductive performance (gestational weight gain, gestation length, number of pups at delivery, pup survival rate at P3). There were no signs of systemic toxicity in *p*-Cresol exposed pups, whose growth curve was undistinguishable from the one of control pups. Although there are not direct reports of *p-*Cresol presence in the developing brain, it is worth noting that the major conjugated form of *p-*Cresol, *p*-Cresyl sulfate, has been previously detected in maternal serum at E14.5^20^. Furthermore, it has been shown that several aromatic metabolites such as hippurate or phenol sulfate, a *p-*Cresyl sulfate parent molecule, are detected in the placenta and/or the embryonic brain, suggesting that aromatic metabolites can cross the placental barrier and reach the embryonic brain^21^. Previous studies have shown that other microbial metabolites, such as 5-aminovalerate, can cross the placental barrier and functionally impact the developing fetal brain^22^. After birth, there is evidence that *p-*Cresol ingested by the mother can be transmitted to pups during lactation, since alkylphenols including *p-*Cresol have been detected in bovine milk^23^. Although we have recently shown that both *p*-Cresol and *p-*Cresyl sulfate can be detected in the adult mouse brain^24^, there is currently no report of *p-*Cresol presence in the developing postnatal brain. However, phenol sulfate is detected early in the postnatal brain at P3 and P21^25^. Given the structural similarity with *p*-Cresyl sulfate, these results suggest that *p-*Cresol can actually reach the developing brain where it can exert neurodevelopmental toxicity. While we cannot completely rule out pup exposure to *p*-Cresol via drinking water, this risk is limited by their physical development. Pups are unable to reach the drinking bottle tip until at least the mid-third week of life, meaning that their primary exposure to *p*-Cresol occurs through maternal milk rather than direct water consumption. Taken together these results suggest that *p-*Cresol ingested by the mother can potentially be transmitted to offspring and reach the developing brain.

We observed mild neurobehavioral effects of *p-*Cresol in the offspring during the early postnatal period. The perinatal exposure to *p-*Cresol did not induce developmental delay in pups as it did not affect key developmental milestones (pinna unfolding, eyelid opening), sensorimotor reflex ontogeny (righting and acoustic startle reflexes), and motor skills (ambulation) acquisition. Also, olfaction and attachment to the mother appeared preserved in the olfactory orientation test. Although we cannot rule out that *p*-Cresol may have a negative impact on maternal behavior and care, which was not assess in the current study, we would likely have observed corresponding effects on pup growth, development, and affective behavior (e.g., olfactory orientation). However, the only deficits observed appeared limited to a modest reduction in USV communication skills at P8 as well as reduced locomotor activity at P12. Regarding USV communication skills at P8, male pups exhibit more noticeable deficits than females at this early stage. However, the early deficits observed in USV communication in male mice pups at P8 could be the precursors of the pronounced social behavior deficits we observe in both male and female adults. Since *p*-Cresol-exposed pups had developed a mature ambulation pattern by P11, similarly to control pups, the reduced locomotor activity at P12 in *p*-Cresol-exposed pups likely indicates reduced exploratory behavior. The impact on locomotor activity appears transient, since it was no longer observed in adulthood in the open-field arena. Finally, perinatal exposure to *p*-Cresol did not affect anxiety levels in adult mice, at least in the non-social setting of the openfield, suggesting that it does not alter emotional processing in adulthood.

In summary, we observed mild neurobehavioral effects of *p-*Cresol in the offspring during the early postnatal period, and no effect on activity or emotional processing in adulthood. However, when looking at behavioral dimensions selectively impaired in ASD patients, *p*-Cresol perinatal exposure induced marked social behavior deficits as well as stereotypic behaviors in adulthood. Given the specificity of the behavioral effects induced by *p*-Cresol exposure, it seems unlikely that *p*-Cresol affects major neurodevelopmental processes, as we would have anticipated much more precocious and broader behavioral effects on offspring. Indeed, neurogenesis starts around E10.5 and is marked by neural progenitor cells in the neuroepithelium starting differentiating to populate the CNS with neurons and glia^26,27^. Defects in neurogenesis would rather cause micro or macrocephaly with global developmental delay associated with broader behavioral impairments^26,27^. Rather, it is possible that *p*-Cresol affects more subtle processes, such as cortical neurons specification and diversity, responsible for the fine-tuning of advanced behaviors such as stereotyped and social behavior^28^.

The current study, combined with our previous work^11^, demonstrates that altered responsiveness to social cues and stereotypic behaviors are hallmarks of *p*-Cresol exposure, regardless of the window of exposure – whether from mid-gestation through lactation (as shown here), or from weaning to adulthood^11^. It would be worth investigating in future studies whether the social behavior deficits and stereotypies induced by *p*-Cresol perinatal exposure appear at adolescence or later in adulthood. While the marked social deficits are of similar magnitude across the exposure windows we have tested here and in^11^, the specific stereotypies induced depend on the timing of exposure. In both our studies we observed an increase in stereotypic circling behaviors. However, unlike our previous study^11^, we found no effect on headshakes. Perinatal *p*-Cresol exposure, however, increased digging behaviors and decreased rearing events. The selective reduction in rearing events could be interpreted as reduced vertical activity, potentially reflecting a residual hypoactivity phenotype observed during the perinatal period. Importantly, extending our previous study, which was conducted only in males^11^, we show that *p*-Cresol exposure affects both males and females, negatively impacting social behavior and inducing certain stereotypic behaviors. This finding contrasts with the reported male-to-female ratio of 3:1 for ASD diagnosis and the higher susceptibility of males to environmental ASD risks^29^. However, the specificity in the observed behavioral changes highlights the potential role of environmental exposures in modulating behaviors impaired in ASD, and suggests that females can be equally sensitive to environmental exposures, depending on the type and timing of exposure.

Environmental exposure to chemical compounds during gestation has raised increasing concerns regarding their developmental neurotoxicity and possible role in neurodevelopmental disorders. The concept of “exposome”—the totality of environmental exposures from conception onwards—has gained significant relevance, especially in relation to neurodevelopmental disorders, including ASD. The perinatal period represents a critical window of neurodevelopment that strongly influences adult behavior^30^. In our study, we demonstrated that perinatal exposure to *p*-Cresol, a metabolite previously linked to ASD, selectively provoked behavioral alterations reminiscent of ASD core symptoms (social deficits, stereotypies). These alterations occurred without affecting overall development, and independently of sex, indicating a specific influence on neurodevelopmental trajectories. The mechanisms underlying the effects of *p-*Cresol on behavior, and more specifically on social behavior, remain to be fully elucidated. Our previous research suggest that postnatal *p-*Cresol exposure impairs the social reward circuit by altering dopamine neurotransmission^11^. Future research is required to confirm whether *p*-Cresol can cross the maternal-fetal barrier and to assess its direct effects on the dopamine system during critical developmental windows.

In conclusion, this study demonstrates that perinatal exposure of mice to *p*-Cresol leads to behavioral alterations reminiscent of core ASD symptoms, including marked deficits in social interaction. Although additional mechanistic studies are required, our findings highlight the potential for early-life exposure to metabolites like *p*-Cresol to contribute to neurodevelopmental disorders. Although a previous study did not find association between *p*-Cresol exposure during pregnancy and increased ASD risk in the human population, the small sample size may have precluded such identification^31^. As stressed earlier, few experimental studies have investigated the causal relationship between pollution exposure on social behavior impairments and there is sufficient evidence to support a critical need for more research^32^. Future epidemiological research focusing on the quantification of *p*-Cresol exposure throughout gestation and in infancy in relation with social abilities in children should also help strengthen the links between *p-*Cresol environmental exposures and social deficits traits in the general population.

## Methods

### Ethics statement for animal housing and experimentation

Animal housing and experimentation were conducted in facilities certified by regional authorities (Direction Départementale de Protection des Populations des Alpes-Maritimes, accreditation #C-06-152-5). The study was in accordance to procedures approved by the local ethics committee for animal experimentation (Ciepal-Azur) and the Ministère de l’Enseignement Supérieur et de la Recherche (APAFIS#19129- 2019062414391395), in agreement with the European Communities Council Directive (2010/63EU) for animal experiments. All methods involving animal experimentation are reported in agreement with ARRIVE guidelines (https://arriveguidelines.org).

### Animals and treatments

Timed-mated C57BL/6J female mice were supplied by Charles River Laboratories (Saint Germain Nuelles, France) at 8.5 days of gestation, corresponding to embryonic day (E) 8.5 post conception. Of note, E0.5 corresponds to the observation of a sperm plug in the morning after overnight mating. Upon arrival, pregnant dams were housed in pairs to reduce stress and promote social interactions. Cages were medium-size open cages filled with wooden bedding, plastic house and enrichment with nesting material, in a temperature (22–24 °C) and hygrometry (70–80%)-controlled room with a 12 h light/dark cycle (lights on from 8:00 a.m. to 8:00 p.m.) with *ad libitum* access to water and food (standard chow, reference 4RF25, Mucedola). At E10.5, *n* = 6 pregnant dams were treated with *p*-Cresol (reference W233706-SAMPLE-K, Sigma-Aldrich), dispensed in sterile drinking water at a concentration of 0.25 g/L as described previously^11^. For the control group, *n* = 5 pregnant dams received water only. Bottles were renewed twice weekly. We minimized disturbances except for weighting dams from E8.5 to E17.5 and changing water bottles and chow twice a week.

To estimate *p*-Cresol intake during pregnancy, water consumption was measured at the cage level. From E10.5 to E14.5, bottles were weighed at E10.5 (9 a.m.) and reweighed at E14.5 (9 a.m.). The difference, divided by 4 days and 2 mice per cage, gave an individual daily intake of 6.03 mL/mouse. Given an average body mass of 28.2 g at E17.5 (Tab. S1), the estimated *p*-Cresol dose was 53 mg/kg/24 h. From E14.5 to E17.5, using the same method, daily drink intake was 5.96 mL/mouse, and at 35.8 g body mass at E17.5 (Tab. S1), the estimated dose was 42 mg/kg/24 h. These values represent average rather than individual doses but remain within the expected 50 mg/kg/24 h range based on a mean body mass of 25 g and a mean drinking water consumption of 5 mL/24 h, used in our previous study^11^.

The day of birth was considered as postnatal day 0 (P0) and the time of birth were recorded. All pups were born between E19-E19.5, ensuring similar prenatal conditions, and the number of pups per litter was recorded at birth (P0) by brief inspection of each cage to minimize maternal stress and perinatal cannibalism which frequently occurs in C57Bl/6J females (Tab. S1). Mothers and pups were left undisturbed until P3, at which point the surviving pups were sexed and the number of male and female recorded (Tab S1). To mitigate litter effects, litters were mixed at P3, and each mother was randomly reassigned 6 to 7 pups to standardize litter size, while maintaining sex balance. This randomized cross-fostering approach prevents the dominance of litter-specific factors (e.g., maternal care variations, genetic influences, intrauterine conditions, restricted milk access in large litters) in driving observed effects. From P3 onwards, each pup was individually labelled on the back using odorless ink markers, with marking renewed every other day to guarantee individual longitudinal follow-up over the course of the experiments. The treatment of mothers was continued after birth throughout lactation and stopped at weaning (P21, the 21st day after birth). At weaning, male pups were separated from female pups, and males and females were housed in independent medium-size (up to 4/5 animals) open cages filled with wooden bedding, plastic house and enrichment with nesting material, and given *ad libitum* access to chow and drinking water.

### Early-life behavioral phenotyping

The acquisition of developmental milestones and reflex ontology, as well as early-life behavior were assessed using procedures derived from Fox’s battery of tests and Wahlsten’s adaption of Fox’s tests, as previously described^33–37^. Pups were tested individually, preferably in the morning (9 a.m.-1 p.m). To limit stress due to maternal separation, the time spent by the pup away from the mother and home cage was limited to the duration of each test. At the end of each test the pup was immediately put back in its home cage.

*Ambulation test*: Ambulation was assessed every day between P7 and P11 to monitor the acquisition of walking proficiency. Each pup was placed on a flat, hard surface and the walking pattern was assessed over 1 min, according to previous scoring criteria^37^: 0 = no movement, 1 = crawling with asymmetric limb movement, 2 = slow crawling but symmetric limb movement, and 3 = fast crawling/walking.

#### Pinna unfolding

The day of the opening of the ear canal, defined as a fully detached outer ear membrane, was recorded in pups aged P3 to P5. A fixed score of 0, 1 or 2 was assigned to each animal, according to the number of ears everted.

#### Eyelid opening

The day of the eyelid opening, defined as any visible break in the membrane covering the eye, was recorded in pups aged P11 to P16. A fixed score of 0, 1 or 2 was assigned to each animal, according to the number of eyelids opened.

#### Righting reflex

Every two days between P4 and P8, each pup was placed on its back on a flat, hard surface, and kept immobile for 5 s. The pup was then released and a hand chronometer started and stopped at the time when the pup returned to the upright position. The time in second was recorded. Animals unable to perform the righting reflex after 1 min were assigned a score of 60 s.

#### Acoustic startle reflex

Pups aged P10 to P16 were examined daily to determine the day of the acoustic startle reflex acquisition known as the Preyer’s reflex which results in a whole body motor response after animals hear a brief loud sound. A metallic cell counter was used to generate the auditive stimulus close to the pup ear (< 5 cm) and the presence (score: 1) or absence (score: 0) of muscle contraction and a startle response was recorded.

#### Ultrasonic Vocalisations (USV) communication

At P8, individual pups were placed on a cotton-padded dish into a thermo-controlled (26 °C) soundproof chamber. USV were recorded for 5 min using the UltraSoundGate Condenser Microphone and 116 USB Audio device (Avisoft Bioacoustics), as described^38,39^. Sonograms were analysed with the AvisoftSASLab Pro software (version 5.2.12, Avisoft Bioacoustics) using a 25 kHz cut-off frequency and a 5–10 ms element separation. USV identified using an automated process embedded in AvisoftSASLab Pro software were then manually curated by removing non-USV signals and adjusting the duration of each USV. After curation, the number and duration of each USV was extracted. We also recorded body temperature before and after USV recording using a thermosensor placed at the nape of the neck.

#### Olfactory Orientation

At P9, each pup was placed into the center of a rectangular-shaped transparent plastic box (15 cm x 30 cm) divided into three zones: the “maternal” bedding on one side, the central zone and the “clean” bedding on the opposite side from the maternal bedding. A Plexiglas odour separator was placed above the central zone to prevent the maternal smell from permeating. The movement of the pup was video recorded over a period of 10 min. The ANY-maze videotracking software was used to determine: the latency to the first entry in the maternal bedding zone and the time spent in each of the three zones.

#### Exploratory behaviour

At P12, each pup was individually placed into a rectangular-shaped transparent plastic box (15 cm x 30 cm). The movement of the pup was recorded over a period of 10 min and the videos were analysed using the ANY-maze videotracking software to determine the total distance travelled and the time spent mobile.

### Adult behavioral phenotyping

Behavioral phenotyping started twelve weeks after weaning. All behavioral experiments were performed one every other day in the following order:

*Dyadic social interactions* Direct social interactions were recorded in four white and opaque quadratic arena (40 cm x 40 cm) with a low light intensity (15 lx). First, the subject mouse was placed in each open-field arena and after 5 s, the subject mouse was put in presence with un unfamiliar sex- and age-matched interactor and their interactions were recorded for 10 min. Manual scoring by an experienced experimenter blind to the experimental group was performed *a posteriori* using an ethological keyboard (Labwatcher^®^, View Point, Lyon, France) by recording the number of nose contacts (nose-to-nose, nose-to-body or nose-to-anogenital region), the time spent in nose contact, and the number of self-grooming events after social contact (corresponding to grooming less than 5 s after social interaction)^11,40,41^. The mean duration of each nose contact was then calculated by dividing the time spent in nose contact by the number of nose contacts.

*Motor stereotypies* Spontaneous stereotyped behaviors were assessed using a protocol adapted from Silverman et al. and Thomas et al.^42,43^. The subject mouse was placed individually in a clean small home cage (40 cm x 11 cm x 17 cm) covered with a thick layer of fresh sawdust (4 cm) and recorded for 10 min with light intensity set at 40 lx. Manual scoring by an experienced experimenter blind to the experimental group was performed *a posteriori* by computing the number of events of rearing, digging, circling episodes, head shakes, grooming and grooming time^44^.

*Open field test* Mice were individually placed in the corner of a white, opaque quadratic arena (40 cm x 40 cm), and their exploration was video-recorded for 10 min under low illumination (15 lx). The center zone of the openfield was defined as a 20 cm x 20 cm square. *A posteriori* videotracking using the ANY-Maze software was used to calculate the total distance travelled (in meters), the number of center entries, the time spent in center, and the mean center velocity (in m.s^− 1^).

### Statistics

*Univariate analyses* To assess the treatment effect between two groups, two-tailed unpaired Mann-Whitney’s U-tests were used due to the small sample size (n **≤** 6 / group). To evaluate the effects of treatment and sex, a two-way analysis of variance (ANOVA) was performed, followed by Sidak’s *post hoc* tests with corrections for multiple comparison. Normality was assessed using Kolmogorov-Smirnov’s test, and non-normally distributed data were log-transformed before conducting ANOVA. For contingency tables analyses, Fisher’s exact test was used. Statistical significance was set at *p* < 0.05 for all tests. Univariate analysis was conducted using GraphPad Prism version 8.00 for Windows (GraphPad Software, USA).

*Modeling of longitudinally collected data* We conducted a multivariable analysis of longitudinally collected developmental and behavioral data using the generalized estimating equations (GEE) approach, which we have previously applied to similar datasets^33^. GEE extends generalized linear models (GLM) to longitudinal data^45^ by estimating the average population response rather than modeling within-individual covariance structures. Unlike standard repeated measures ANOVA, which is primarily applied to normally distributed continuous data and has limited capacity to handle more than two factors, GEE accounts for correlations between repeated measures within the same individual and adjusts for the random effects of multiple covariates which can be continuous, binary, or count data. In our analysis, GEE was used to model each repeatedly measured developmental and behavioral outcome, with treatment, sex, and developmental time included as covariates^45,46^. Notably, GEE effectively captured the global effects of these variables on developmental or behavioral outcomes. GEE was implemented using the R Package *geepack*^47^.

## Electronic supplementary material

Below is the link to the electronic supplementary material.

Supplementary Material 1

## Data Availability

The datasets generated and analyzed during the current study will be made available by the corresponding author upon reasonable request.
