# Supplementary material for: Perinatal exposure to the autism-linked metabolite p-Cresol has limited impact on early development in mice but lasting effects on adult social behavior
Source: Sci Rep. 2025 Apr 15;15:12934. doi: 10.1038/s41598-025-96840-8 (PMC12000446; doi:10.1038/s41598-025-96840-8)
Supplement: Supplementary file 1 — Supplementary Material 1 [file 41598_2025_96840_MOESM1_ESM.pdf]

Perinatal exposure to the autism-linked metabolite *p*-Cresol has limited impact on early development in mice but lasting effects on adult social behavior

Juliette Canaguier, Geoffroy Mallaret, Cristina Paraschivescu, Julie Le Merrer, Jérôme Becker, Nicolas Glaichenhaus, Laetitia Davidovic

File content:

- Supplementary table 1
- Supplementary figures (3)
- Supplementary figures legends (3)

		Body mass (g)		Pups # at P0	Surviving pups # at P3		Litter composition at P3		Sex ratio at P3		Cohort composition at P3	
**Treatment**	**Female ID**	**E13.5**	**E17.5**	**Total #**	**Total #**	**Survival rate (%)**	**Female #**	**Male #**	**Female %**	**Male %**	**Female #**	**Male #**
**Control**	1	30.8	39.1	7	7	100.0%	4	3	57.1%	42.9%		
	2	27.2	33.5	8	6	75.0%	1	5	16.7%	83.3%		
	3	28.1	35.8	8	7	87.5%	1	6	14.3%	85.7%	11	12
	4	31.5	38.6	8	6	75.0%	3	3	50.0%	50.0%		
	5	26.8	33.4	8	7	87.5%	2	5	28.6%	71.4%		
	Mean	***28.9***	***36.1***	***7.8***	***6.6***	***84.6%***	***2.2***	***4.4***	***33.3%***	***66.7%***		
***p*-Cresol**	6	27.1	33.9	7	7	100.0%	4	3	57.1%	42.9%		
	7	28.1	33.4	8	6	75.0%	4	2	66.7%	33.3%		
	8	26.1	33.6	7	7	100.0%	4	3	57.1%	42.9%		
	9	30.4	39.8	8	6	75.0%	3	3	50.0%	50.0%	22	18
	10	27.4	35.5	8	7	87.5%	3	4	42.9%	57.1%		
	11	29.9	38.8	7	7	100.0%	4	3	57.1%	42.9%		
	Mean	***28.4***	***36.2***	***7.5***	***6.7***	***88.9%***	***3.7***	***3.0***	***55.0%***	***45.0%***		
***p*-Cresol vs Control comparison**		**ns[a]**	**ns[a]**	**ns[a]**	**ns[a]**	-	-	-	-		**ns[b]**	

Supplemental Table S1. Control and *p*-Cresol-exposed litters characteristics at birth (P0) and prior randomized cross-fostering at P3.

[a] Mann & Whitney U-test, ns: $p>0.05$; [b] Fisher's exact test, ns: $p>0.05$; -: no statistical test was performed.

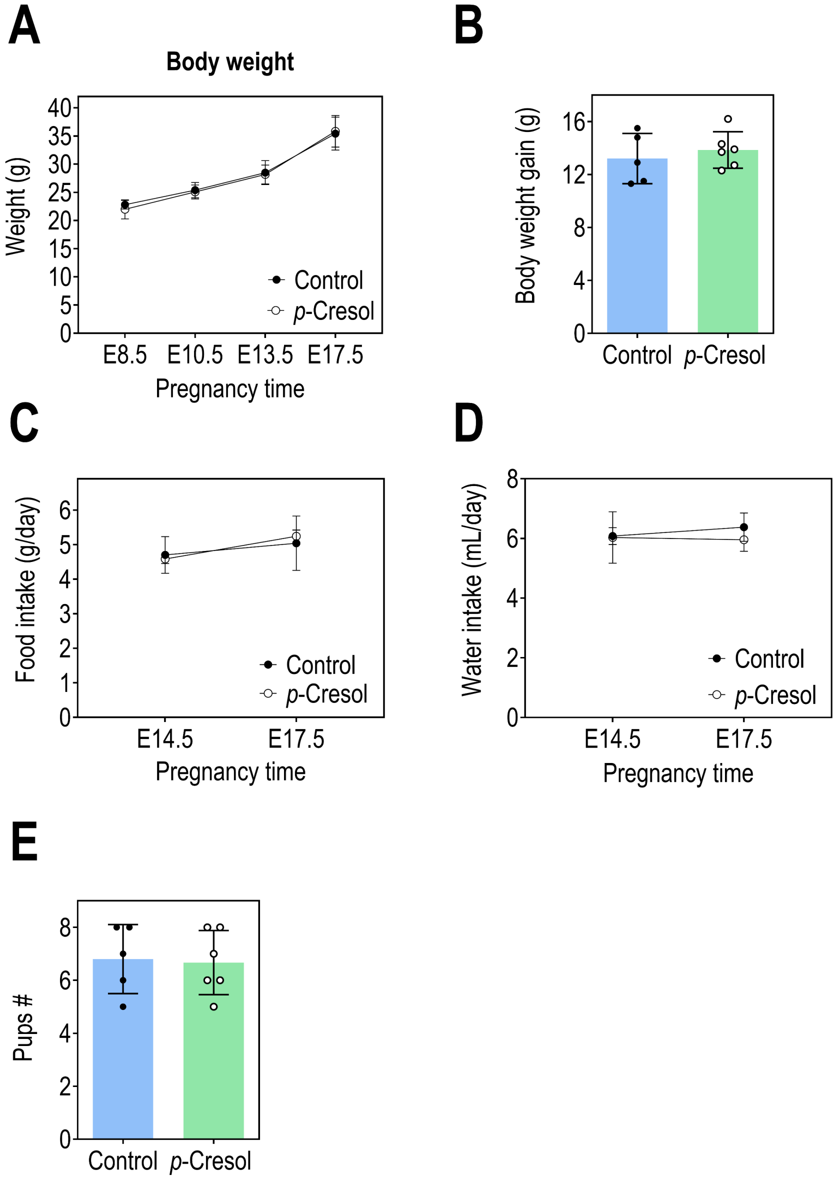

Supplemental Figure 1. Global health parameters and pregnancy outcome of control or *p*-Cresol-treated mice.

A. Body weight (E8.5 to E17.5); two-way ANOVA: p(Treatment) = 0.7979, p(Time) < 0.0001, p(Treatment x Time) = 0.5052; Šidák post hoc tests for treatment effect : p > 0.05.

B. Body weight gain (E8.5 to E17.5); Mann-Whitney U-test: p > 0.05.

C. Food intake (E14.5 to P2) two-way ANOVA: p(Treatment) = 0.9936, p(Time) < 0.0001, p(Treatment x Time) = 0.2198; Šidák post hoc tests for treatment effect : p > 0.05.

D. Drink intake (E14.5 to E17.5) two-way ANOVA: p(Treatment) = 0.3500, p(Time) = 0.5938, p(Treatment x Time) = 0.3836; Šidák post hoc tests for treatment effect : p > 0.05.

E. Number of pups born (P0) and surviving at P3 from control and *p*-Cresol treated dams; Mann-Whitney U-test: p > 0.05.

A, C, D. Data are presented as means ± standard deviation. **B, E.** Data are presented as dot plots with means ± standard deviation.

A-E. n=5 control and n=6 *p*-Cresol-treated mice.

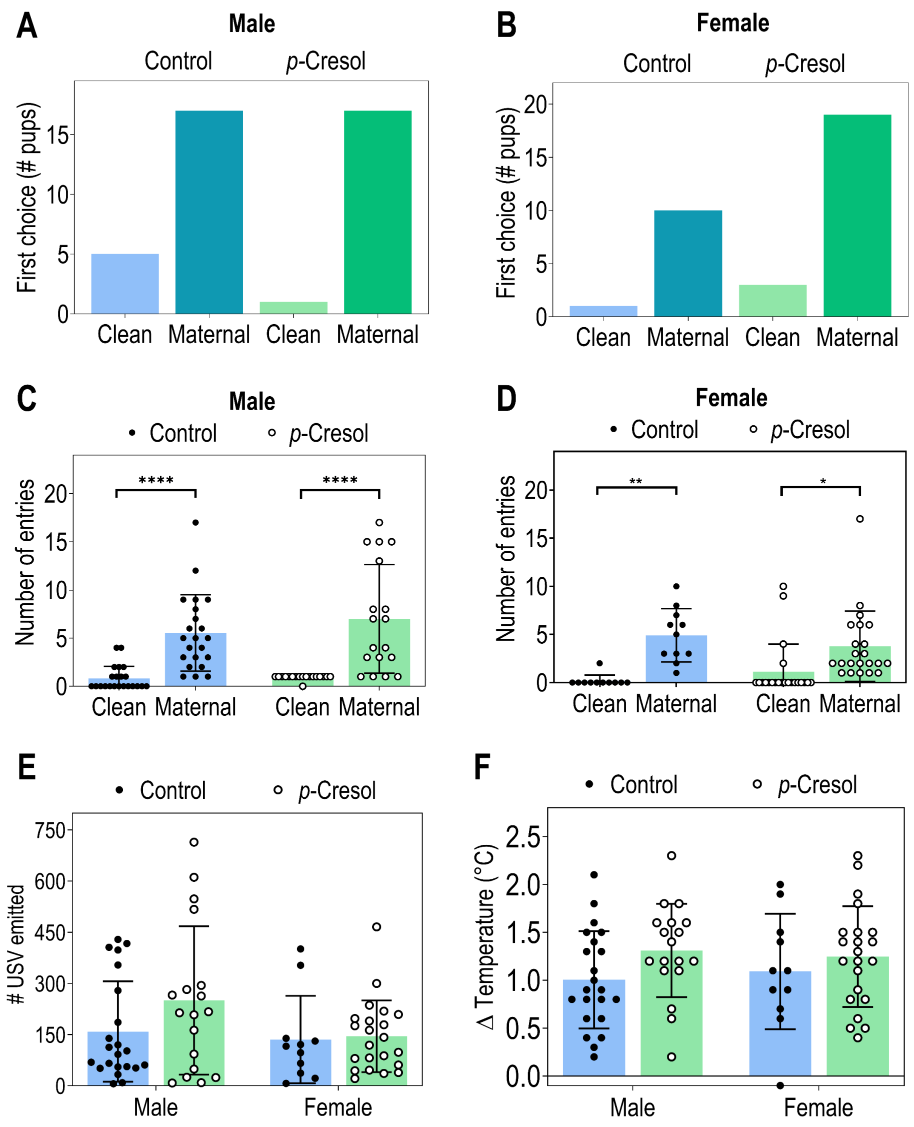

Supplemental Figure 2. Additional behavioral parameters for olfactory orientation and USV monitoring in pups and adult control and *p*-Cresol treated mice

A. First zone chosen by each male pup; Fisher's exact test: $p = 0.1969$.

B. First zone chosen by each female pup; Fisher's exact test: $p > 0.9999$.

C. Number of entries in each zone for males pups; two-way ANOVA: p(Treatment) = 0.3301, p(Zone) < 0.0001, p(Treatment x Zone) = 0.3800; Šidák post hoc tests for zone effect: ****p < 0.0001.

D. Number of entries in each zone for females pups; two-way ANOVA: p(Treatment) = 0.9013, p(Zone) < 0.0001, p(Treatment x Zone) = 0.2021; Šidák post hoc tests for zone effect: *p < 0.05, **p < 0.01.

E. Total number of USV emitted by male and female pups; two-way ANOVA: p(Treatment) = 0.1847, p(Sex) = 0.0930, p(Treatment x Sex) = 0.2815; Šidák post hoc tests for treatment effect by sex: p > 0.05.

F. Change in pup's body temperature during USV recording; two-way ANOVA: p(Treatment) = 0.0748, p(Sex) = 0.9355, p(Treatment x Sex) = 0.5529; Šidák post hoc tests for treatment effect by sex: p > 0.05.

A, B. Data are represented as a diagram indicating the number of choices of the zone by group. C, D, E, F: Data are presented as dot plots with means ± standard deviation. *p < 0.05, **p < 0.01, ****p < 0.0001. Only statistically significant differences are presented (p < 0.05)**.**

A-F. n = 33 control (22 males/11 females), n = 40 *p*-Cresol (18 males/22 females).

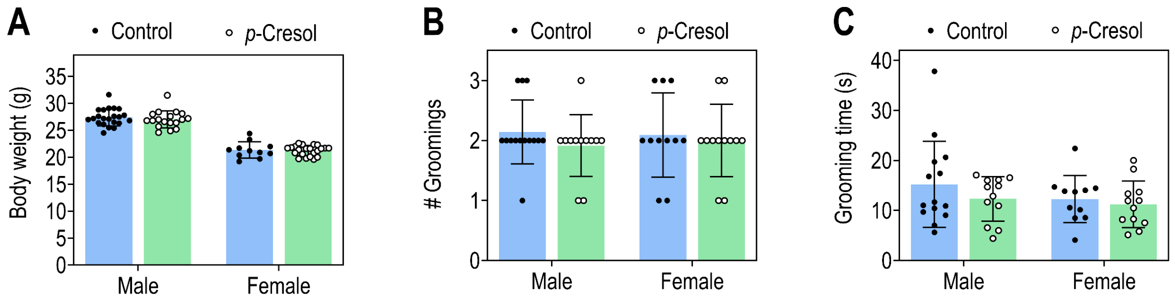

Supplemental Figure. 3 Perinatal exposure to *p*-Cresol does not alter body weight and grooming behavior in adult mice.

A. Body weight at 3.5 month; two-way ANOVA: p(Treatment) = 0.4838, p(Sex) < 0.0001, p(Treatment x Sex) = 0.7020; Šidák post hoc tests for treatment effect : p > 0.05.

B. Number of groomings; two-way ANOVA: p(Treatment) = 0.3519, p(Sex) = 0.9262, p(Treatment x Sex) = 0.6901; Šidák post hoc tests for treatment effect by sex: p > 0.05.

C. Time spent in grooming; two-way ANOVA: p(Treatment) = 0.2618, p(Sex) = 0.2504, p(Treatment x Sex) = 0.5994; Šidák post hoc tests for treatment effect by sex: p > 0.05.

A-C. Data are presented as dot plots with means ± standard deviation.

A-C. n = 25 control (14 males/11 females), n = 24 *p*-Cresol (12 males/12 females).
